# Flux Balance Analysis of Plant Metabolism: The Effect of Biomass Composition and Model Structure on Model Predictions

**DOI:** 10.3389/fpls.2016.00537

**Published:** 2016-04-26

**Authors:** Huili Yuan, C. Y. Maurice Cheung, Peter A. J. Hilbers, Natal A. W. van Riel

**Affiliations:** ^1^Department of Biomedical Engineering, Eindhoven University of TechnologyEindhoven, Netherlands; ^2^Yale-NUS CollegeSingapore, Singapore; ^3^Institute for Complex Molecular Systems, Eindhoven University of TechnologyEindhoven, Netherlands

**Keywords:** biomass composition, model structure, sensitivity, flux balance analysis, central carbon metabolism, *Arabidopsis*, large-scale metabolic model

## Abstract

The biomass composition represented in constraint-based metabolic models is a key component for predicting cellular metabolism using flux balance analysis (FBA). Despite major advances in analytical technologies, it is often challenging to obtain a detailed composition of all major biomass components experimentally. Studies examining the influence of the biomass composition on the predictions of metabolic models have so far mostly been done on models of microorganisms. Little is known about the impact of varying biomass composition on flux prediction in FBA models of plants, whose metabolism is very versatile and complex because of the presence of multiple subcellular compartments. Also, the published metabolic models of plants differ in size and complexity. In this study, we examined the sensitivity of the predicted fluxes of plant metabolic models to biomass composition and model structure. These questions were addressed by evaluating the sensitivity of predictions of growth rates and central carbon metabolic fluxes to varying biomass compositions in three different genome-/large-scale metabolic models of *Arabidopsis thaliana*. Our results showed that fluxes through the central carbon metabolism were robust to changes in biomass composition. Nevertheless, comparisons between the predictions from three models using identical modeling constraints and objective function showed that model predictions were sensitive to the structure of the models, highlighting large discrepancies between the published models.

## Introduction

Flux balance analysis (FBA), a constraint-based modeling approach, is widely used in predicting metabolic fluxes based on stoichiometric metabolic models, in particular, large-scale or genome-scale metabolic models (GSMs; Orth et al., 2010). Stoichiometric metabolic models are typically underdetermined because the number of reactions in the model is usually larger than the number of metabolites (Bonarius et al., 1997; Kauffman et al., 2003). Therefore, in most cases, constraint-based analysis yields multiple feasible flux solutions. To narrow down the space of feasible solutions, additional constraints can be imposed by specifying the range of fluxes through any particular reaction. In addition to the application of constraints, an objective function is usually defined for identifying biologically relevant flux solutions. The most commonly used objective function for FBA is the biomass objective function (BOF), which is to maximize the efficiency of biomass production, i.e., growth rate (Feist and Palsson, 2010). Biomass production is mathematically represented by a so called “biomass reaction” which, in essence, is a collection of all individual biomass constituents together with their fractional contributions to the overall cellular biomass, and energetic requirements for the biomass generation.

Knowledge of the biomass composition is crucial for predicting flux distribution in metabolic models using FBA because the intracellular fluxes are dependent on the fluxes contributing to biomass synthesis (Pramanik and Keasling, 1997; Schwender and Hay, 2012). Therefore, an important consideration during the development of GSMs is to define the biomass composition, ideally for the condition under study. Experimental evidence indicates that the biomass composition varies between species, cell types and physiological conditions (Novak and Loubiere, 2000; Hay and Schwender, 2011). However, due to a lack of organism-specific and/or condition-specific experimental information, the biomass compositions used in plant GSMs are often collected from diverse types of measurements, experiments, research groups, and even different cell types and plant species (Collakova et al., 2012).

Some computational methods have been developed to estimate the fractional contribution of a precursor to the biomass reaction in microorganisms, e.g., calculating the coefficients of deoxy-nucleotide triphosphates (dNTPs) and nucleotide triphosphates (NTPs) according to the fraction of DNA and RNA (Thiele and Palsson, 2010). Nevertheless, these approaches can only be used for amino acids, NTPs (ATP, GTP, CTP, UTP), and dNTPs (dATP, dGTP, dCTP, dTTP). Given the existence of multiple organelles in plants, one needs to be cautious when applying these approaches to plant models.

With the increasing use of network reconstructions and constraint-based approaches, a need has arisen to clearly define and demonstrate the relevance of the modeling parameters, such as biomass composition, for predicting metabolic fluxes. Work examining the influence of the biomass composition on the predicted fluxes has mostly been done on models of microorganisms, in particular *Escherichia coli* (Pramanik and Keasling, 1997; Feist et al., 2007). Most recently, a sensitivity analysis of a yeast model suggested that model predictions are sensitive to variations in biomass composition (Dikicioglu et al., 2015). These observations naturally lead to questions about the sensitivity of flux predictions in plant metabolic networks to biomass composition and the robustness of plant metabolic models. Thus far, there are very limited studies exploring the effects of changes in biomass composition on the flux distributions in plants. Plant metabolic networks are significantly more complex than those of microorganisms due to the presence of multiple compartments and parallel metabolic pathways. A study on a model of oilseed rape suggested that flux predictions are sensitive to the contents of oil and protein, which are the major storage components in oil seed (Schwender and Hay, 2012). However, in a study of Arabidopsis heterotrophic cell culture, central carbon metabolism has been observed to be robust to different conditions despite the significant differences in the resulting biomass compositions (Williams et al., 2010). Given that plants are adapted to grow in diverse environmental conditions, plant metabolism is expected to be flexible in face of perturbations. Thus, it deserves theoretical exploration on basis of constraint-based metabolic models to assess the influence of changing biomass composition on predicted fluxes.

Arabidopsis, a model organism for plant biology, has been studied extensively with systems-biology approaches. In this study, we started by reviewing the published Arabidopsis metabolic models followed by an investigation of the impact of changing the biomass composition on the flux predictions in large- or genome-scale plant metabolic models, in particular, the fluxes through central metabolic pathways [i.e., glycolysis, pentose phosphate pathway (PPP), TCA cycle, and mitochondrial electron transport chain (ETC)]. This is because these existing large-scale metabolic networks of plants provided mostly qualitative predictions of intracellular fluxes for primarily central carbon metabolism. Furthermore, previous work has shown that fluxes of central carbon metabolism dominate the FBA results, with little to no flux through the secondary metabolic pathways (Collakova et al., 2012). Here, we focused on study on three published models of Arabidopsis, which have different biomass compositions and network structures. We systematically evaluated the influence of biomass composition on the outcome of FBA simulations in three ways: (1) using different biomass compositions with the same model; (2) using the same biomass composition with different models; (3) varying individual components of the biomass composition and maintenance cost. Our analyses indicate that (i) the central metabolic fluxes are relatively stable in face of varying biomass composition, regardless of model structure; and (ii) the model structure is the main factor in determining the variation in computational results generated by using FBA.

## Methods

### Stoichiometric models

In this study, we compared and investigated three published stoichiometric models of Arabidopsis, denoted as Poolman (Poolman et al., 2009), AraGEM (de Oliveira Dal'Molin et al., 2010a), and AraCore (Arnold and Nikoloski, 2014). The Systems Biology Markup Language (SBML) format for the Poolman and AraCore model were available from supplementary files of the corresponding paper. The direction of the phenylpyruvate carboxylase reaction in the Poolman model has been corrected as reported in their subsequent publication (Williams et al., 2010). For the AraGEM model, an updated version was obtained from http://web.aibn.uq.edu.au/cssb/resources/Genomes.html. In this study, we simulated the cellular metabolism of Arabidopsis cells growing on glucose as carbon and energy sources under aerobic heterotrophic conditions.

Although all major biomass components (i.e., cell wall, protein, lipid, carbohydrate, DNA, and RNA) were taken into account in the three models via representative metabolites or corresponding precursors, the biomass components included in these three models are not exactly the same. For example, xylose and γ-aminobutyric acid (GABA) were not considered in Poolman model, but were included in AraGEM and AraCore models. Similarly, soluble metabolites were only considered in AraCore model, but not in Poolman and AraGEM models. To keep the list of biomass components as consistent as possible between the three models, we added some additional biomass transporters to the models if required. It is noted that not all newly added biomass components can be produced by all models, e.g., maltose, xylose, and GABA cannot be produced in Poolman model, coniferyl-alcohol, coumaryl-alchol, sinapyl-alcohol, and xylose cannot be produced by AraCore model as these metabolites are not present in AraCore. Therefore, in this study, we only added new biomass components that can be produced by all the three Arabidopsis models. In total, we added nine new biomass transporters to Poolman model and seven for AraGEM (Supplementary Data 1).

### Biomass equations

**Figure 1:**
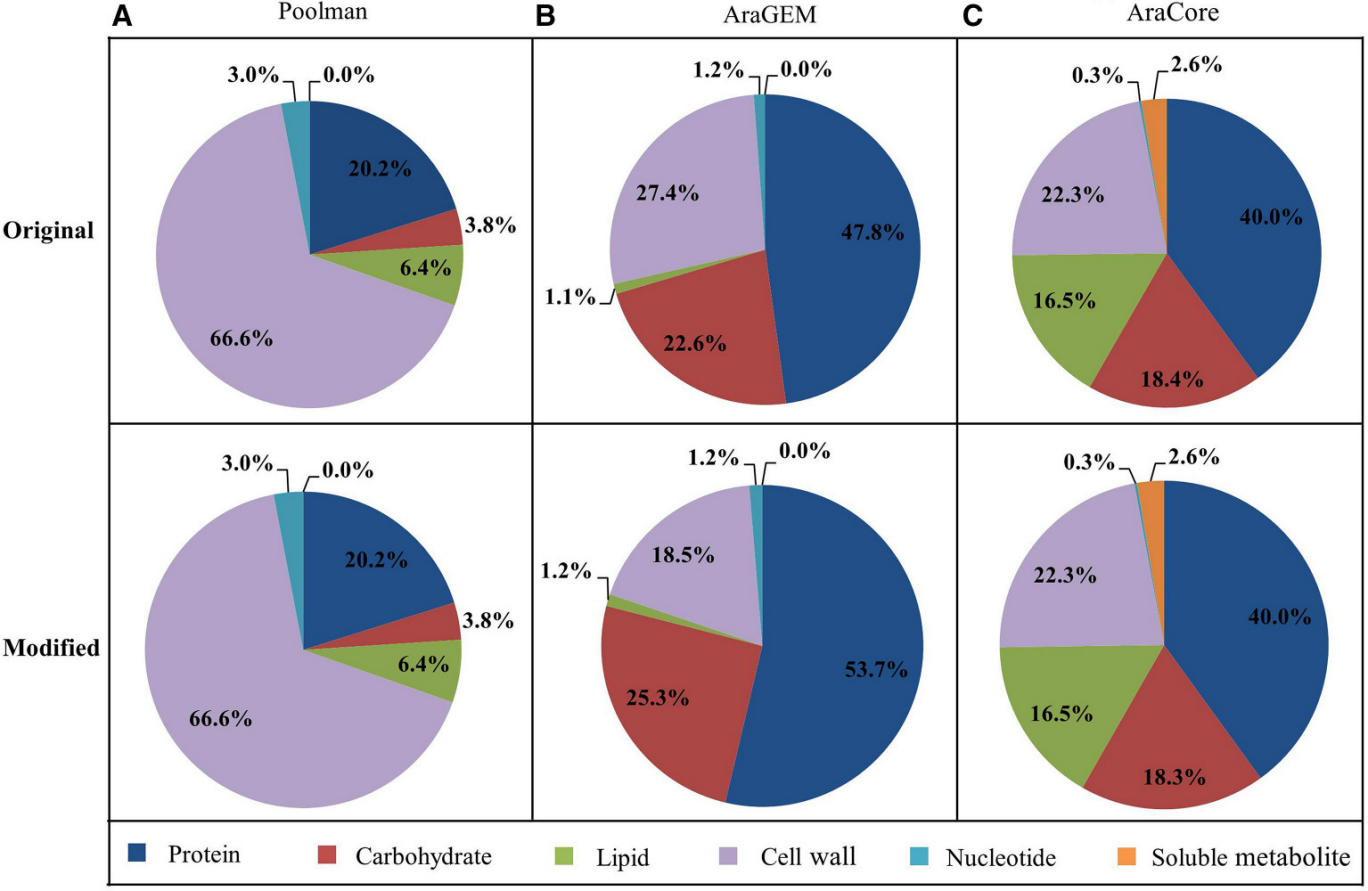
**Weight percentage of the biomass components**. The original and modified (used for simulations) weight percentage for each class of metabolites contributing to biomass synthesis is displayed. The composition is displayed for Poolman model **(A)**, AraGEM model **(B)**, and AraCore model **(C)**. The calculations for each class of metabolites are shown in Supplementary Data 2.

The biomass compositions used in this study were extracted from previous studies, hereafter referred to as PoolmanBOF, AraGEMBOF, and AraCoreBOF, corresponding to the biomass composition used in Poolman, AraGEM and AraCore model, respectively (Supplementary Data 2). Since three condition-specific biomass compositions (reflecting carbon-limiting, nitrogen-limiting, and optimal growth conditions) have been employed in the AraCore model, here we chose the carbon-limiting biomass reaction to represent AraCoreBOF. The original biomass composition of each model, together with the calculation of weight percentage of biomass components (Figure 1) is provided in Supplementary Data 2.

Due to the different units originally used in the three studies (mmol/g/L in Poolman and mmol/g DW in AraGEM and AraCore), the biomass compositions were normalized to enable a fair comparison. These calculations were performed by weight, defining 1 unit of flux through the biomass equation equals to 1 g of biomass. The details of these calculations are provided in Supplementary Data 2. Finally, we used the normalized biomass equations to perform our model simulations.

### Model simulations

FBA was used to determine the flux solutions at steady-state condition. All simulations were performed using FBA in geometric mode (Smallbone and Simeonidis, 2009) as implemented in the COBRA toolbox (Becker et al., 2007) executed in MATLAB (The MathWorks, version R2012a). Geometric FBA enables a unique optimal solution that is central to the range of possible flux distributions. The Gurobi Optimizer (http://www.gurobi.com, version 5.0.2) solver in combination with the COBRA toolbox were used to solve the linear programming problems. In our study, the biomass equation was maximized to obtain the optimal solution of the metabolic model as described elsewhere (Orth et al., 2010). Formally, the FBA problem can be stated as follows:

$$\begin{array}{ll} Maximize:{\text{v}}_{\text{growth}}\; & \;\\ \underset{i}{\sum }{\text{c}}_{i}{\text{X}}_{i}+GAM\overset{v_{growth}}{\to }1Biomass\; & \; \end{array}$$

$$\begin{array}{lll} \text{Subject to } & \text{Sv}=0\; & \;\\ \text{and } & {\text{v}}_{\text{min}}\leq \text{v}\leq {\text{v}}_{\text{max}}\; & \; \end{array}$$

where ***v***_***growth***_ is the flux that the biomass reaction carries, representing growth rate, *c* is the vector of biomass coefficients, whose component ***c***_*i*_ indicates the ratio of *v* metabolite ***X***_*i*_ required for the formation of a unit of biomass, ***S*** is the stoichiometric matrix, is a vector of all reaction fluxes in the system, also referred to as the flux distribution, ***v***_***min***_ and ***v***_***max***_ represent lower and upper bounds for the flux of each reaction, respectively. GAM refers to growth associated maintenance.

To simulate the cellular behavior of Arabidopsis cells, we constrained the glucose uptake rates at 10 flux units, which was the only source of carbon and energy. AraCore represents a photoautotrophic cell, which does not have organic sources for heterotrophic scenarios, but it can also be utilized to simulate heterotrophic conditions by adapting the energy source (Arnold and Nikoloski, 2014). Consequently, we added an additional glucose exchange reaction “Im_Glc” to AraCore model. Ammonia (NH_3_) and hydrogen sulfide (H_2_S) were constrained to be utilized as the sole N and S sources, respectively, because AraGEM and AraCore can only grow with H_2_S and NH_3_ as the S and N sources, respectively. Since SO${}_{4}^{2-}$ is the sole S source in the Poolman model, it does not have an H_2_S transporter. We therefore added an H_2_S transporter to Poolman model, namely “H2S_tx”, enabling H_2_S as sole sulfur source in all models. P_*i*_ is the sole P source input in all three models. Additionally, non-growth associated maintenance (NGAM) was included in all simulations with a value of 2.02, which is a normalized flux unit referring to the value reported in Poolman model (Supplementary Data 2). Similarly, GAM was fixed at 53.26 flux units in this study, which was scaled based on the value reported in AraGEM model. Imported or exported metabolites are always freely exchangeable across the system boundary to provide the necessary nutrients and remove secreted substances.

The biomass equation (i.e., BOF) is generated by defining all of the biomass constituents, in which all the precursor metabolites are assembled in one single reaction with corresponding coefficients (Feist and Palsson, 2010; Thiele and Palsson, 2010). For a fair comparison, we only considered the biomass components which can be produced by all three models in the three biomass equations (Supplementary Data 2). This results in new biomass compositions for AraGEM and AraCore models because some biomass metabolites such as xylose and maltose that cannot be produced by all three models are not taken into account in our biomass reaction. The modified weight percentages of biomass compositions that are included in our biomass reactions differs slightly from the original ones for the three models (Figure 1).

To investigate the influence of biomass composition on the predicted fluxes, we performed three scenarios in each model with the biomass equations of PoolmanBOF, AraGEMBOF, and AraCoreBOF, respectively. We replaced the coefficient of each biomass component in the biomass equation with the corresponding values in the other two models. In total, nine scenarios were simulated in the study, namely “Poolman-PoolmanBOF,” “Poolman-AraGEMBOF,” “Poolman-AraCoreBOF,” “AraGEM-PoolmanBOF,” “AraGEM-AraGEMBOF,” “AraGEM-AraCoreBOF,” “AraCore-PoolmanBOF,” “AraCore-AraGEMBOF,” and “AraCore-AraCoreBOF.” To assess the differences between predicted fluxes obtained from different scenarios, we define the biomass composition given in each model as the “reference” scenario. Thus, “Poolman-PoolmanBOF,” “AraGEM-AraGEMBOF,” and “AraCore-AraCoreBOF” are the “reference” scenarios in Poolman, AraGEM, and AraCore model, respectively.

To confirm the confidence of the predictions, flux variability analysis (FVA) was conducted for the nine model-biomass combinations, which determines the range of possible solutions for each reaction while giving rise to the same optimal value for the objective function (Mahadevan and Schilling, 2003).

## Results

### Comparisons between plant flux-balanced models

#### Origins and uses of biomass compositions in plant flux-balanced models

To understand how the biomass composition data is obtained in published metabolic models, we surveyed the source of data used in formulating biomass equations in the existing large-scale metabolic models of plants (Table 1). From the survey, it was verified that only 5 out of 21 models had their biomass compositions measured by the research group that constructed the model, whereas, the remaining 16 studies either do not include any biomass information or adopted from other research groups, some of which were from other organisms. Furthermore, within the surveyed plant metabolic networks, 6 of 21 (29%) used biomass data as the objective function, 11 of 21 (52%) used biomass data as constraints, whereas the rest did not perform any FBA simulations.

**Table 1:** **Summary of the origin of biomass data in the existing large-scale metabolic models of plants**.

**Species**	**No**.	**Model**	**Biomass composition data**	**Biomass used as**
Arabidopsis *(Arabidopsis thaliana)*	1	Poolman et al., 2009	Experimental	Constraint
	2	de Oliveira Dal'Molin et al., 2010a	Literature (Guinn, 1966; Poorter and Bergkotte, 1992; Niemann et al., 1995)	Constraint
	3	Radrich et al., 2010	No biomass	No simulation
	4	Saha et al., 2011	Literature (Spector, 1956; Muller et al., 1970; Penningd et al., 1974; Wedig et al., 1987) or other related organisms	No simulation
	5	Mintz-Oron et al., 2012	Literature (Weise et al., 2000; Reinders et al., 2005; Poolman et al., 2009; de Oliveira Dal'Molin et al., 2010a)	No simulation
	6	Chung et al., 2013	No biomass	No simulation
	7	Cheung et al., 2013	Experimental	Constraint
	8	Arnold and Nikoloski, 2014	Literature (Döermann et al., 1995; Sharrock and Clack, 2002; Mooney et al., 2006; DeBolt et al., 2009; Tschoep et al., 2009; Pyl et al., 2012; Sulpice et al., 2013)	Objective
Barley *(Hordeum vulgare)*	9	(Grafahrend-Belau et al., 2009)	Literature (OECD, 2004)	Objective
	10	Grafahrend-Belau et al., 2013	Literature (Antongiovanni and Sargentini, 1991; Bonnett and Incoll, 1993a,b)	Constraint
Rapeseed (*Brassica napus*)	11	Hay and Schwender, 2011	Biomass macromolecules determined experimentally, the composition of biomass macromolecules obtained from literature (Katterman and Ergle, 1966; Norton, 1989; Schwender and Ohlrogge, 2002; Town et al., 2006)	Constraint
	12	Pilalis et al., 2011	Literature (Schwender et al., 2004, 2006)	Objective
Maize (*Zea mays*)	13	de Oliveira Dal'Molin et al., 2010b	Literature (Poorter and Bergkotte, 1992; Niemann et al., 1995; Guinn, 1966)	Constraint
	14	Saha et al., 2011	Literature (Spector, 1956; Muller et al., 1970; Penningd et al., 1974; Wedig et al., 1987) or other related organisms	Objective
	15	Simons et al., 2014	Experimental	Objective
Sorghum *(Sorghum bicolor)*	16	de Oliveira Dal'Molin et al., 2010b	Literature (Guinn, 1966; Poorter and Bergkotte, 1992; Niemann et al., 1995)	Constraint
Sugarcane *(Saccharum officinarum)*	17	de Oliveira Dal'Molin et al., 2010b	Literature (Guinn, 1966; Poorter and Bergkotte, 1992; Niemann et al., 1995)	Constraint
Rice (*Oryza sativa*)	18	(Poolman et al., 2013)	Literature (Juliano, 1985; Kwon and Soh, 1985)	Constraint
	19	Lakshmanan et al., 2013	Literature (Juliano, 1985; Edwards et al., 2012)	Objective
Tomato *(Solanum lycopersicum)*	20	Colombié et al., 2015	Experimental	Constraint
	21	Yuan et al., 2016	Literature (Sheen, 1983; Roessner-Tunali et al., 2003; Schauer et al., 2005; Nunes-Nesi et al., 2007; Sánchez-Rodríguez et al., 2010; El-Sayed, 2013)	Constraint

#### General properties of published arabidopsis flux-balanced models

To investigate the variability of the biomass compositions and structure of the models for the same species, we chose Arabidopsis since eight metabolic models were published since 2009. The general statistics of the available Arabidopsis models to date is summarized in Table 2. In general, we observed an increase in the number of genes, metabolites, reactions, and transporters included in Arabidopsis metabolic models over time, but this increase was not uniform. For example, the number of metabolites and reactions in the model of Mintz-Oron et al. (2012) is much larger than that of AraGEM (de Oliveira Dal'Molin et al., 2010a), but the latter has more genes than the former. Toward the compartmentalization, the organelles included in each model are relatively invariant over time. The various Arabidopsis genome-scale models were reviewed more extensively elsewhere (Collakova et al., 2012; de Oliveira Dal'Molin and Nielsen, 2013; Baghalian et al., 2014; Arnold et al., 2015).

**Table 2:** **Structure comparison of the reconstructed metabolic models for Arabidopsis**.

**References**	**Abbreviation**	**Year**	**Organelles**	**Number of genes**	**Number of metabolites**	**Number of reactions**	**Number of exchange reactions**	**Number of intracellular transporters**
Poolman et al., 2009	Poolman^[a]^	2009	2 (c,m)	Not available	1253	1406	42	–
de Oliveira Dal'Molin et al., 2010a	AraGEM^[a]^	2010	5 (c,m,p,x,v)	1419	1737	1601	18	81
Radrich et al., 2010	Radrich^[a]^	2010	–	1571	2328	2315	–	–
Saha et al., 2011	*i*RS1597^[a]^	2011	5 (c,m,p,x,v)	1597	1820	1844	18	81
Mintz-Oron et al., 2012	Mintz-Oron^[a]^	2012	7 (c,m,p,x,v,g,e)	1223	2930	3508	101	772
Chung et al., 2013	*i*AT1475^[*b*]^	2013	4 (c,m,p,x)	1475	1761	1895	22	86
Cheung et al., 2013	Cheung^[a]^	2013	5 (c,m,p,x,v)	2857	2739	2769	20	192
Arnold and Nikoloski, 2014	AraCore^[a]^	2014	4 (c,m,p,x)	634	407	549	98	124

Symbol “–“indicates information is unknown; “^a^” indicates information is extracted from the original models; “^b^” indicates information is retrieved from papers. Exchange reactions allow for exchange of specific metabolites with the extracellular space. Transporters indicate metabolites can move between intracellular organelles; “c,” cytosol; “m,” mitochondria; “p,” plastid; “x,” peroxisome; “v,” vacuole; “g,” Golgi; “e,” endoplasmic reticulum.

Besides the general statistics, these models differ in several aspects, for instance, the inclusion of cellular maintenance costs (Table 3). The models are quite different regarding cell maintenance, because its experimental quantification is a major challenge (Sweetlove et al., 2013).

**Table 3:** **Network characteristics and FBA simulations in Arabidopsis GSMs**.

**Items model**	**Cell type**	**Model format**	**Cell maintenance**	**Objective function**	**BOF included**
Poolman	Heterotrophic	ScrumPy and SBML	NGAM	Minimize total flux	PoolmanBOF
AraGEM	Photosynthetic and Heterotrophic	SBML	GAM	Minimize photon/sucrose uptake of growth rate	AraGEMBOF
Radrich	Unknown	SBML	Not included	Not included	Not included
*i*RS1597	Photosynthetic and Heterotrophic	Excel	GAM	Maximize biomass	AraGEMBOF
Mintz-Oron	Photosynthetic and Heterotrophic	SBML	GAM	Minimize metabolic adjustment (MOMA)	AraGEMBOF
*i*AT1475	Photosynthetic and Heterotrophic	Excel	GAM	Maximize IPP production	AraGEMBOF
Cheung	Photosynthetic and Heterotrophic	ScrumPy and SBML	GAM and NGAM	Five objective functions^[a]^	Biomass as constraints
AraCore	Photosynthetic and Heterotrophic	SBML	Not included	Maximize biomass and energy efficiency	AraCoreBOF

“PoolmanBOF” indicates the biomass objective function included in the Poolman model; “AraGEMBOF” indicates the biomass objective function included in the AraGEM model; “AraCoreBOF” indicates the biomass objective function included in the AraCore model. GAM, growth associated maintenance; NGAM, non-growth associated maintenance. ^[a]^5 objective functions are minimization of overall flux, maximization of biomass, minimization of glucose consumption, maximization of ATP production and maximization of NADPH production.

#### Three arabidopsis models have different biomass compositions

Poolman, AraGEM, and AraCore models each use a different biomass composition to simulate cell growth. Although the relative amounts of each biomass component for all three models were derived from experimental data, they relied entirely on different sources. Poolman model used measurements from their own group for modeling a heterotrophic cell culture, while, AraGEM and AraCore used data for various tissues for Arabidopsis or related species. As a further comparison, we analyzed the macromolecular compositions for each model (Supplementary Data 2), finding the three models differ significantly in the composition of biomass macromolecules (Figure 1). In Poolman model, cell wall comprised more than half of cell biomass. In contrast, cells contain higher amounts of protein in AraGEM and AraCore model. Experimental evidence indicates that the distributions of biomass components are very tissue-specific (Ohlrogge and Browse, 1995; Mueller et al., 2003). One would expect that the biomass compositions of AraGEM and AraCore to be similar as both models represent photosynthetic leaf cells. While there are some similarities in terms of the proportions of cell wall, carbohydrate, and protein, there are also major differences. For example, the proportion of lipid in AraCore biomass is much larger than in AraGEM biomass (18.4 vs. 1.1%). This could be explained by the fact that biomass data in AraGEM model is collected from different organisms.

#### Differences in central carbon metabolism between the three arabidopsis models

Going beyond merely comparing general network characteristics, we compared the models at the individual reaction level, concentrating on the compartmentation and reversibility of reactions in central carbon metabolism, which is an essential biological process to sustain growth and biomass synthesis (Supplementary Figure 1). We focused on three Arabidopsis models, Poolman, AraGEM, and AraCore models, which have distinct model structures and biomass compositions. Generally, the three models covered all the listed central metabolic reactions (Supplementary Table 1). Compared with the other two models, AraGEM did not include PPi-dependent phosphofructokinase (EC 2.7.1.90), NADP-dependent non-phosphorylating glyceraldehyde-3-phosphate dehydrogenase (EC 1.2.1.9) or NAD-dependent 6-phosphogluconate dehydrogenase (EC 1.1.1.343). Moreover, AraGEM uses two lumped reactions to represent the electron transport chain (ETC) reactions, which are referred to as alternative oxidase pathway (AOX) and cytochrome C oxidase pathway (COX). In contrast, Poolman and AraCore models incorporate separate reactions to describe the ETC. Beyond these differences, the localization and directionality of central metabolic reactions in the three models are not always consistent. For example, the reaction catalyzed by phospho*enol*pyruvate carboxylase (EC 4.1.1.31) in Poolman model operates in the opposite direction compared to other two models, which was corrected in a subsequent publication (Williams et al., 2010). For AraCore, some reactions known to be reversible, such as aconitase (EC 4.2.1.3) and fumarase (EC 4.2.1.2), are set as irreversible. Poolman model is not fully compartmentalized, so most of the reactions involved in central carbon metabolic pathways are assigned to the cytosol.

### The impact of biomass composition and model structure on central metabolic fluxes

#### Central carbon metabolism is robust to changes in biomass composition

**Figure 2:**
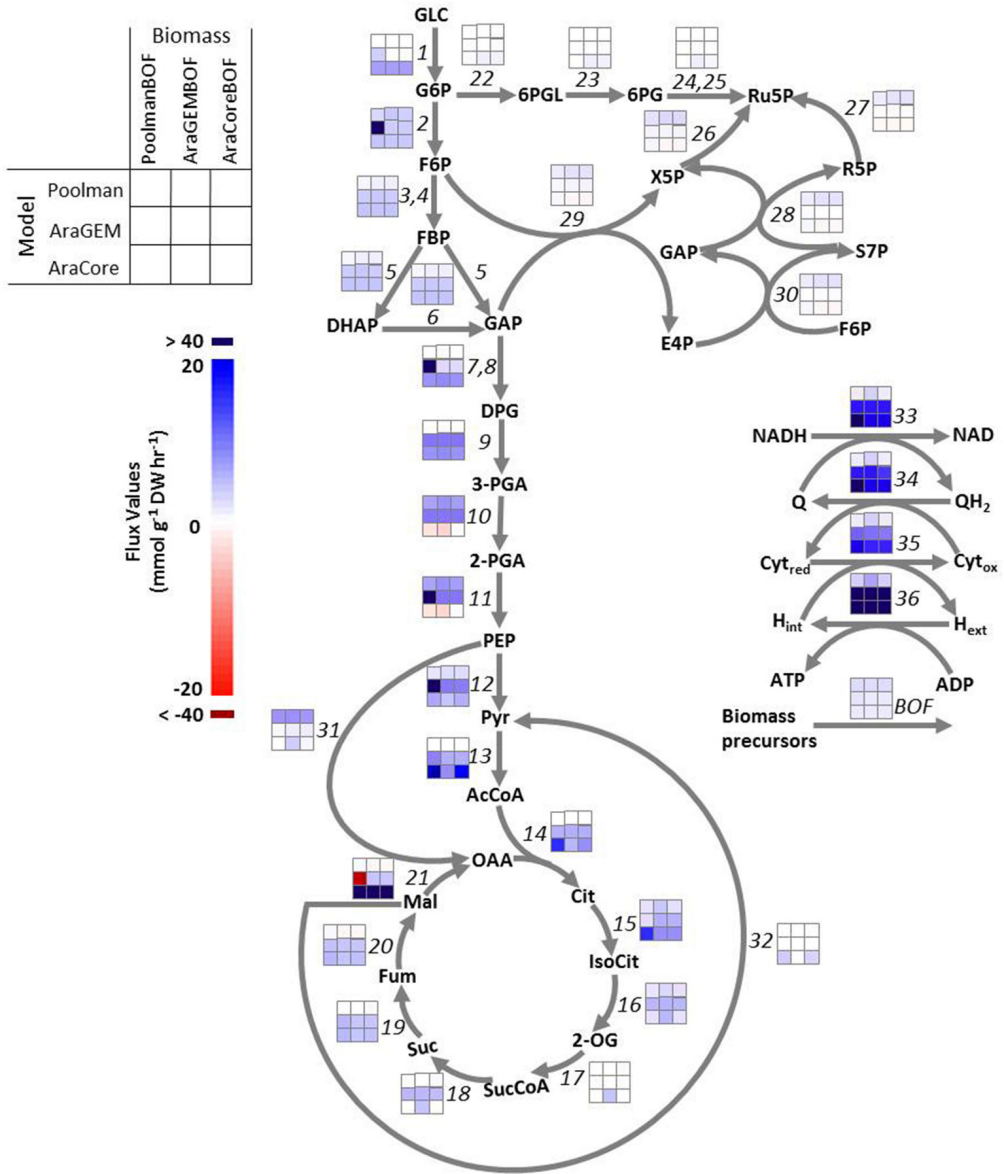
**Flux maps of central carbon metabolism predicted from three Arabidopsis models: Poolman model, AraGEM model, and AraCore model**. Fluxes were predicted using three different biomass compositions in each model: PoolmanBOF, the biomass composition included in Poolman model; AraGEMBOF, biomass composition included in AraGEM model; AraCoreBOF, biomass composition included in AraCore model. Each reaction is numbered, referencing Table S1, and the color intensity of each box corresponds to the flux value (mmol g^−1^ DW h^−1^) for the respective labeled reaction in each scenario. The results calculated by different biomass compositions with the same model can be interpreted by comparing between columns (compare horizontally). The results calculated by the same biomass composition with different models can be interpreted by comparing between rows (compare vertically). DW, Dry cell weight. Metabolite abbreviations are as follows: GLC, glucose; 2-OG, 2-oxoglutarate; 3-PGA, 3-phosphoglycerate; 2-PG, 2-phosphoglycolate; G6P, glucose-6-phosphate; F6P, fructose-6-phosphate; 6PGL, 6-phosphogluconolactone; 6PG, 6-phosphogluconate; Ru5P, ribulose-5-phosphate; R5P, ribose-5-phosphate; X5P, xylulose-5-phosphate; S7P, sedoheptulose-7-phosphate; E4P, erythrose-4-phosphate; FBP, fructose-1,6-biphosphate; DHAP, dihydroxyacetone phosphate; DPG, glycerate-1,3-bisphosphate; PEP, phosphoenol pyruvate; OAA, oxalacetic acid; GAP, glyceraldehyde-3-phosphate; 2-PGA, 2-phosphoglycerate; Pyr, pyruvate; Cit, citrate; IsoCit, *threo*-isocitrate; Suc, succinate; SucCoA, succinyl-CoA; Fum, fumarate; Mal, malate; QH2, ubiquinone; Q, ubiquinol; Cyt_*red*_, cytochrome reduced; Cyt_*ox*_, cytochrome oxidized; Fd_*red*_, ferredoxin reduced; Fd_*ox*_, ferredoxin oxidized.

To test the sensitivity of FBA solutions to the biomass composition, nine scenarios described in the Methods Section were simulated with three flux-balanced metabolic models of Arabidopsis, Poolman, AraGEM, and AraCore models, in combination with their respective biomass compositions. The maximization of growth rate is used as the objective function subjecting to mass-balance constraints, and setting the glucose uptake rates as 10 flux units (“Methods”). In particular, we analyzed how central metabolic reactions listed in Supplementary Table 1 respond to a change in biomass composition. The results are illustrated in Figure 2 in which the differences in color intensity between columns (compare horizontally) reflects the differences in flux values of each reaction calculated by the three biomass compositions. It can be seen that for the majority of the reactions, in particular the glycolytic reactions, the flux patterns were very similar. This indicates a high stability of the central carbon metabolism in Arabidopsis with respect to biomass composition, regardless of model structures.

Some reactions showed larger flexibility in our predictions. The flux distribution predicted by AraCore model with AraGEMBOF resulted in flux through 2-oxoglutarate dehydrogenase reaction (reaction 17), a reaction belonging to the TCA cycle, whereas this reaction did not carry flux with PoolmanBOF and AraCoreBOF. The flux distributions through the TCA cycle reflect the function of the metabolic network, and its operation largely depends on the cell type and the considered physiological context (Sweetlove et al., 2010). In addition, 2-oxoglutarate (2-OG) is an essential intermediate for the biosynthesis of amino acids such as glutamine and glutamate (Supplementary Figure 1). Given that AraGEMBOF contains a much higher amount of glutamine and glutamate (Supplementary Data 2), it is not surprising that 2-oxoglutarate dehydrogenase reaction carried non-zero flux implemented with AraGEMBOF in AraCore model. For AraGEM model, the biomass composition greatly affected the fluxes through glyceraldehyde-3-phosphate dehydrogenase (EC 1.2.1.9/1.2.1.12; reaction 7 and 8), phosphoglycerate kinase (EC 2.7.2.3; reaction 9), phosphoglycerate hydratase (EC 4.2.1.11; reaction 11), pyruvate kinase (EC 2.7.1.40; reaction 12) and malate dehydrogenase (EC 1.1.1.37; reaction 21). For example, the use of PoolmanBOF on the AraGEM model gave rise to higher fluxes through malate dehydrogenase compared to using AraGEMBOF, which is due to a reaction cycle that malate dehydrogenase involves in with PoolmanBOF. Commonly, the great changes occurred at the branch points of glycolysis and the TCA cycle, where there were drains for the synthesis of cellular constituents. Under heterotrophic conditions, it is generally thought that the oxidative PPP (OPPP) predominately provides reducing power for the production of biomass, in particular for fatty acid synthesis. As a result, we would expect the OPPP reactions to be active in our simulations. However, OPPP reactions carried no flux in any of the predicted solutions of Poolman and AraGEM model. Interestingly, for AraCore model, using AraGEMBOF resulted in small flux through OPPP reactions such as glucose 6-phosphate (EC 1.1.1.49; reaction 22), 6-phosphogluconolactonase (EC 3.1.1.31; reaction 23) and 6-phosphogluconate dehydrogenase (EC 1.1.1.44/1.1.1.343; reaction 24, 25). Previously, Williams et al. (2010) showed that the OPPP was poorly predicted by Poolman model, which was suggested to be caused by the error in assigning the reversibility of the NADP^+^-dependent glyceraldehyde-3-phosphate dehydrogenase (EC 1.2.1.13; Cheung et al., 2013).

#### Model structure has a large impact on model predictions

Furthermore, we assessed the effects of model structures on the flux predictions by comparing the model predictions from the same biomass equation with different models (Figure 2, comparisons between rows; compare vertically). It is apparent that the vast majority of central metabolic reactions carried different fluxes from each other, despite the application of identical biomass composition and boundary constraints. For example, the hexokinase reaction (reaction 1), which converts glucose to glucose-6-phosphate for entry into glycolysis, carried significantly different fluxes in three Arabidopsis models, irrespective of the biomass equation used. Based on 10 flux units of glucose, the Poolman model produces 2.84 units of biomass by using the biomass equation of Poolman model (i.e., PoolmanBOF), while the models of AraGEM and AraCore synthesize 1.53 and 1.42 biomass units respectively. Similarly, Poolman model yields the highest biomass by using AraGEMBOF and AraCoreBOF (2.40 and 2.12 flux units, respectively), while, AraGEM generates the lowest biomass (1.29 and 1.13 flux units, respectively; Figure 2). This indicates that the Poolman model predicts more efficient conversion of glucose into biomass compared to the other two models. Overall, model structure has a large impact on the model prediction made with FBA, which highlights the importance of the quality of metabolic models on the variation of model predictions.

To quantify the variation of fluxes in face of changing biomass composition and model structure, we calculated the standard deviation, a robust measure for the dispersion within a set of data, for each reaction fluxes that were predicted from nine model-biomass equation combinations. We then compared the median of the standard deviation (Supplementary Data 3). The medians of the standard deviations of reaction fluxes for the cases with the same biomass composition but different models (4.59, 3.35, and 3.22 for PoolmanBOF, AraGEMBOF, and AraCoreBOF combinations, respectively) were systematically higher than that of simulations with the same metabolic network (0.29, 0.53, and 1.98 for Poolman, AraGEM, and AraCore combinations). This indicates that the predicted fluxes were considerably more dispersed when using different models, despite of the same biomass equation, revealing that model structure has a larger impact on the FBA predictions.

For further confirmation, FVA was performed to examine the flux capacity for each reaction under the 9 model-biomass combinations (Supplemental Data 4). FVA analysis showed that 10 of 33 considered reactions (pyruvate dehydrogenase, reaction 13; citrate synthase, reaction 14; succinate thiokinase, reaction 18; complex II, reaction 19; glucose 6-phosphate dehydrogenase, reaction 22; 6-phosphogluconolactonase, reaction 23; ribulose-phosphate 3-epimerase, reaction 26; ribulose 5-phosphate epimerase, reaction 27; transketolase 1, reaction 28; and transketolase 2, reaction 29) have non-overlapping flux variability ranges in the compared scenarios and agreed with the FBA analysis. However, the rest have a large range of possible flux values in, at least one of considered scenarios, resulting in overlapping flux variability ranges in the compared scenarios, which are not comparable.

### Robustness of growth rate predictions with respect to changes in individual biomass component and maintenance cost

From Figure 2, we observed that growth rate is not sensitive to the biomass composition. Considering the BOF is the employed objective in this study, which is the most commonly used objective for FBA (Feist and Palsson, 2010), we further analyzed the impact of changing the fractional contribution of each single biomass component on the growth rates with the “reference” scenarios—Poolman-PoolmanBOF, AraGEM-AraGEMBOF, and AraCore-AraCoreBOF three scenarios. The coefficient of each compound in the biomass equation was independently varied 30% up or down (this value referred to Pinchuk et al., 2010) in each “reference” scenario, while the composition of the rest of the biomass components was kept unchanged. The resulting model predictions of the growth rates were compared with that of “reference” biomass equation. Overall, our analyses showed that the predicted growth rates were not sensitive to changes in the ratios of biomass components. For instance, growth rates in AraGEM and AraCore models were altered by, at most, 4 and 6%, respectively (Supplementary Tables 3, 4). The only exception is in the Poolman model where a 30% decrease or increase of the composition of cell wall led to 19.9% and 14.3% variation in growth rate, respectively (Supplementary Table 2). This discrepancy is not unexpected as cell wall is the largest part (66.6%) of the overall biomass composition in the Poolman model (Figure 1).

Our analysis preferentially revealed that growth rate has a larger change at higher coefficient in terms of C atom. Given that glucose, which is the carbon and energy source, was used as the limiting nutrient in the analyses in this study, we would expect growth rate correlates to the fractional coefficient of C atom. To quantify this effect, we calculated the Pearson correlation coefficient (PCC), a measure of the linear dependence between two variables, for all predicted growth rates simulated by decreasing and increasing of each single biomass component by 30% (Supplementary Figure 2). We also calculated the correlation coefficients between changes in growth rate and the coefficient in terms of weight (Supplementary Figure 3). We found that the correlations among the coefficient in terms of weight were systematically similar to the observed correlations among the coefficient in terms of C atoms (Supplementary Figure 2), indicating that when the fractional contribution of C atoms were altered, the growth rates were increased or decreased in a synchronized fashion.

In addition to the structure of the metabolic network and biomass composition, the *in silico* growth rate can also be influenced by the maintenance cost, i.e., GAM and NGAM. Therefore, we evaluated the influence of maintenance on the predicted growth rates with three “reference” scenarios characterized by constraints on: (i) no maintenance, (ii) sole GAM, (iii) sole NGAM, and (iv) GAM and NGAM. We then compared the growth rates of scenario (i), (ii), and (iii) with that of scenario (iv), separately. The analyses showed that the influence of maintenance parameters varies with the used model. The exclusion of GAM and/or NGAM changes the growth rate by, at most, 29.6 and 1.3% in AraGEM model and AraCore model, respectively (Supplementary Table 5). Given that AraGEM and AraCore models describe the ETC reactions quite differently (Section Differences in Central Carbon Metabolism between the Three Arabidopsis Models), it is unsurprising to observe large discrepancies between the influence of maintenance cost on growth rate predictions for these two models. It is worth mentioning that the growth rate predicted from the Poolman model did not change with the maintenance, which is biologically implausible. This is because there exist futile cycles in Poolman model (Arnold et al., 2015).

## Discussion

In this study, we investigated the effect of biomass composition and model structure on steady-state flux predictions in the metabolic models of Arabidopsis. Different combinations (three models and three biomass equations) were tested to cover multiple possibilities for biomass composition. Our results demonstrated that flux predictions of the central metabolic network are fairly insensitive to changes in biomass composition, regardless of the employed metabolic networks (Figure 2). Biomass demands are a principal drive of the flux distributions in metabolic models, therefore one would expect the flux distributions to be sensitive to changes in the biomass composition. However, several experimental studies provided evidence that central carbon metabolism is rather stable when the substrate source remains unchanged (Rontein et al., 2002; Spielbauer et al., 2006; Junker et al., 2007; Williams et al., 2008, 2010). In a study of heterotrophic Arabidopsis cell culture, fluxes through the central metabolic network measured by metabolic flux analysis (MFA) were observed to be unchanged under different physiological conditions, despite under which the biomass composition altered (Williams et al., 2008). Consistently, Spielbauer et al. (2006) found that flux distributions of central carbon metabolism were stable in maize endosperm. Nonetheless, under conditions with different substrates provided, the flux distribution in the central carbon metabolism altered significantly (Junker et al., 2007). The noticeable stability of central carbon metabolism has been extensively demonstrated in many microorganism studies (Pramanik and Keasling, 1997, 1998; Feist et al., 2007).

Our results also showed that 30% variations in individual biomass precursor had a minor effect on the growth rates with Arabidopsis models (Supplementary Tables 2–4). This observation is in agreement with the previously reported findings (Pramanik and Keasling, 1998; Puchalka et al., 2008). Puchalka et al. (2008) reported that varying a single biomass component by 20% up or down has a negligible effect (< 1%) on the growth rate. Due to the large number of macromolecules such as protein and carbohydrate in the cell, it is not surprising that single biomass component, which makes up the macromolecules did not significantly affect the growth rate. However, in other studies, it was found that varying the macromolecular composition had considerable effect on the flux simulations (Feist et al., 2007; Nookaew et al., 2008). Similarly, our analyses showed that the effect of maintenance cost on growth rate was limited (Supplementary Table 5), which contradicts previous results of *E. coli* model that maintenance cost had larger impact on growth rate prediction (Feist et al., 2007). However, it is important to point out that the growth yield altered merely by 5% when increasing or decreasing GAM twofold in the study of Puchalka et al. (2008), whereas, the effect of NGAM depends on the rate of carbon source supply. The discrepancies of the effect of maintenance cost on growth rate predictions of models of different organisms is likely to due to the difference in lifestyle and metabolic behavior of the different organisms.

Sensitivity analyses of biomass composition performed on different models often yields conflicting results. According to the sensitivity analysis on the *E. coli* model of Feist et al. (2007), small changes in biomass composition did not significantly affect growth rates, while, cellular maintenance cost can considerably influence growth rate prediction. However, the study of the *E. coli* model of Varma and Palsson (1994) noted that predictions of constraint-based model are not very sensitive to maintenance, including GAM and NGAM. Recently, Dikicioglu et al. (2015) found that the predicted fluxes of the metabolic models of yeast were sensitive to biomass composition. These are likely caused by inherent differences in the respective metabolic networks. To verify this, we examined the differences of FBA solutions between different models by constraining the same biomass outputs and cellular maintenance costs, and our results indicated that model structure has a larger impact on the flux predictions than the biomass composition (Figure 2). Moreover, our analyses of the impact of maintenance on the predicted growth rates showed large differences between the three examined models that represent the ETC reaction differently (Supplementary Table 5). These observations indicate the structure of metabolic models is a major determinant of the variation in model predications.

Although, our analysis indicated the robustness of the growth rate and central metabolic fluxes in response to changes in biomass composition and maintenance cost, it does not suggest that it is sufficient to generalize biomass composition from any cell type within a plant or from other closely related organisms. This is because the biomass composition for different tissues and organisms may include metabolites specific to them, which need specific determination. Notably, variables other than model structure, such as the P/O ratio have considerable impacts on flux distributions according to previous reports (Feist et al., 2007).

## Conclusions

In this study, we reviewed the characteristics of published Arabidopsis metabolic models, concentrated on three particular models with distinct model structures and different compositions of biomass components. We examined the sensitivity of predicted fluxes to biomass composition, which is particularly relevant to plant metabolic models for which the biomass data is often collected from various sources. Our analysis showed that central metabolic fluxes as well as growth rates were insensitive to the variations in biomass composition, but were significantly affected by model structure. This work represents a thorough set of analyses performed in plants by means of constraint-based modeling, thereby providing relevant information about how critical FBA solutions can be affected by biomass composition, and more importantly by the structure of the models. Despite differences in several aspects such as model structure and number of metabolites and reactions included, each of the evaluated models has its own merits. Comparative analysis of the models paves the way for exploring the existence of principles that are relevant for the regulation and robustness of plant central carbon metabolism.

## Author contributions

HY, CC, PH, and NV conceived and designed the work. HY carried out all simulations and drafted the manuscript. CC, PH, and NV revised the manuscript. All authors read and approved the final version of the manuscript.

### Conflict of interest statement

The authors declare that the research was conducted in the absence of any commercial or financial relationships that could be construed as a potential conflict of interest.
