# Supplementary material for: Flux Balance Analysis of Plant Metabolism: The Effect of Biomass Composition and Model Structure on Model Predictions
Source: Front Plant Sci. 2016 Apr 26;7:537. doi: 10.3389/fpls.2016.00537 (PMC4845513; doi:10.3389/fpls.2016.00537)
Supplement: Supplementary file 1 [file Table1.DOCX]

Table S1. Central carbon metabolic reactions included in three Arabidopsis models. Each reaction is numbered, referencing Figure S1. ‘->’ indicates the irreversibility of a reaction from left to right; ‘<=>’ indicates the reversibility of a reaction. The abbreviation ahead of an arrow indicate the compartment in which the corresponding reaction occurs, ‘c’ refers to cytosol, ‘p’ refers to plastid, ‘m’ refers to mitochondria.

| **Reaction Number** | **Enzyme Name** | **EC** | **Compartmentation and reversibility** | | |
| --- | --- | --- | --- | --- | --- |
|  |  |  | **Poolman** | **AraGEM** | **AraCore** |
|  | ***Glycolysis*** |  |  |  |  |
| 1 | Hexokinase | 2.7.1.2 | c(->) | c(->) | c(->) |
| 2 | Phosphoglucose isomerase | 5.3.1.9 | c(<=>) | c/p(<=>) | c/p(<=>) |
| 3 | Phosphofructokinase (ATP)**^[1]^** | 2.7.1.11 | c(->) | c/p(->) | c/p(->) |
| 4 | Phosphofructokinase (PPi) | 2.7.1.90 | c(->) | Not included | c(<=>) |
| 5 | Aldolase | 4.1.2.13 | c(<=>) | c/p(<=>) | c/p(<=>) |
| 6 | Triose phosphate isomerase | 5.3.1.1 | c(<=>) | c/p(<=>) | c/p(<=>) |
| 7 | Glyceraldehyde-3-phosphate dehydrogenase (NADP^+^) | 1.2.1.9 | c(->) | Not included | c(->) |
| 8 | Glyceraldehyde-3-phosphate dehydrogenase (NAD^+^) | 1.2.1.12 | c(->) | c/p(<=>) | c/p(<=>) |
| 9 | Phosphoglycerate kinase | 2.7.2.3 | c(->) | c/p(<=>) | c/p(<=>) |
| 10 | Phosphoglycerate mutase | 5.4.2.1 | c(<=>) | c/p(<=>) | c/p(<=>) |
| 11 | Phosphoglycerate hydratase | 4.2.1.11 | c(<=>) | c/p(<=>) | c/p(<=>) |
| 12 | Pyruvate kinase | 2.7.1.40 | c(<=>) | c/p(->) | c/p(->) |
|  | ***TCA cycle*** |  |  |  |  |
| 13 | Pyruvate dehydrogenase | 1.8.1.4/1.2.4.1/  2.3.1.12 | m(->) | m(->) | m(->) |
| 14 | Citrate synthase | 2.3.3.1 | m(<=>) | m(->) | m(->) |
| 15 | Aconitase | 4.2.1.3 | m(<=>) | m(<=>) | m(->) |
| 16 | Isocitrate dehydrogenase | 1.1.1.41 | m(<=>) | m(<=>) | m(->) |
| 17 | 2-oxoglutarate dehydrogenase | 1.2.4.2/1.8.1.4 | m(<=>) | m(<=>) | m(->) |
| 18 | Succinate thiokinase | 6.2.1.5 | m(<=>) | m(<=>) | m(->) |
| 19 | Complex II | 1.3.99.1/1.3.5.1 | m(->) | m(<=>) | m(->) |
| 20 | Fumarase | 4.2.1.2 | m(<=>) | m(<=>) | m(->) |
| 21 | Malate dehydrogenase | 1.1.1.37 | m(<=>) | m(<=>) | m(<=>) |
|  | ***Pentose Phosphate Pathway*** |  |  |  |  |
| 22 | Glucose 6-phosphate dehydrogenase | 1.1.1.49 | c(->) | c/p(->) | p(->) |
| 23 | 6-Phosphogluconolactonase | 3.1.1.31 | c(<=>) | c/p(<=>) | p(->) |
| 24 | 6-Phosphogluconate dehydrogenase (NADP^+^) | 1.1.1.44 | c(<=>) | c/p(->) | p(<=>) |
| 25 | 6-Phosphogluconate dehydrogenase (NAD^+^) | 1.1.1.343 | c(<=>) | Not included | p(<=>) |
| 26 | Ribulose -phosphate 3-epimerase | 5.1.3.1 | c(<=>) | c/p(<=>) | p(<=>) |
| 27 | Ribose 5-phosphate epimerase | 5.3.1.6 | c(<=>) | c/p(<=>) | p(<=>) |
| 28 | Transketolase 1 | 2.2.1.1 | c(<=>) | c/p(<=>) | p(<=>) |
| 29 | Transketolase 2 | 2.2.1.1 | c(<=>) | c/p(<=>) | p(<=>) |
| 30 | Transaldolase | 2.2.1.2 | c(<=>) | c/p(<=>) | p(<=>) |
|  | ***Others*** |  |  |  |  |
| 31 | PEP carboxylase | 4.1.1.31 | **c(<-)** | c(<=>) | c(<=>) |
| 32 | Malic enzyme(NADP^+^) | 1.1.1.40 | c(->) | p(->) | c/p(->) |
| 33 | Complex I | 1.6.5.3 | m(->) | m(->) | m(->) |
| 34 | Complex III | 1.10.2.2 | m(->) | m(->) | m(->) |
| 35 | Complex IV | 1.9.3.1 | m(->) | m(->) | m(->) |
| 36 | Complex V | 3.6.3.14 | m(->) | m(->) | m(<=>) |

**^[1]^** specifies the type of the enzyme involved in the respective reaction.
