# Supplementary material for: Flux Balance Analysis of Plant Metabolism: The Effect of Biomass Composition and Model Structure on Model Predictions
Source: Front Plant Sci. 2016 Apr 26;7:537. doi: 10.3389/fpls.2016.00537 (PMC4845513; doi:10.3389/fpls.2016.00537)
Supplement: Supplementary file 2 [file Table2.DOCX]

Table S2. Influence of individual biomass component on the growth rate predicted from Poolman model in the ‘Poolman-PoolmanBOF’ scenario. The growth rate was predicted by varying single biomass components up or down by 30%.

| **Compound** | **% Change in growth rate** | | **Coefficient** | **MW** | **Empirical formula** | **C atoms** |
| --- | --- | --- | --- | --- | --- | --- |
|  | **-30%** | **30%** |  |  |  |  |
| Cellulose | 19.93 | -14.25 | 1.947989 | 180 | C_6_H_12_O_6_ | 6 |
| Palmitate | 3.69 | -3.44 | 0.250407 | 256 | C_16_H_32_O_2_ | 16 |
| Alanine | 0.99 | -0.97 | 0.2746215 | 89 | C_3_H_7_NO_2_ | 3 |
| Starch | 0.96 | -0.94 | 0.111102 | 180 | C_6_H_12_O_6_ | 6 |
| Leucine | 0.95 | -0.93 | 0.1059743 | 131 | C_6_H_13_NO_2_ | 6 |
| Glutamine | 0.83 | -0.82 | 0.1546882 | 146 | C_5_H_10_N_2_O_3_ | 5 |
| Glutamate | 0.82 | -0.81 | 0.152979 | 146 | C_5_H_8_NO_4_ | 5 |
| Serine | 0.58 | -0.58 | 0.1632345 | 105 | C_3_H_7_NO_3_ | 3 |
| Asparagine | 0.53 | -0.52 | 0.1475663 | 132 | C_4_H_8_N_2_O_3_ | 4 |
| Phenylalanine | 0.52 | -0.51 | 0.0401677 | 165 | C_9_H_11_NO_2_ | 9 |
| Aspartate | 0.51 | -0.51 | 0.1432931 | 132 | C_4_H_6_NO_4_ | 4 |
| Threonine | 0.43 | -0.42 | 0.1196483 | 119 | C_4_H_9_NO_3_ | 4 |
| Isoleucine | 0.38 | -0.38 | 0.0535569 | 131 | C_6_H_13_NO_2_ | 6 |
| Valine | 0.25 | -0.25 | 0.0356096 | 117 | C_5_H_11_NO_2_ | 5 |
| Tyrosine | 0.25 | -0.24 | 0.0344701 | 181 | C_9_H_11_NO_3_ | 9 |
| Histidine | 0.20 | -0.19 | 0.0219355 | 155 | C_6_H_9_N_3_O_2_ | 6 |
| Arginine | 0.18 | -0.18 | 0.0336155 | 175 | C_6_H_15_N_4_O_2_ | 6 |
| Glycine | 0.15 | -0.15 | 0.0851782 | 75 | C_2_H_5_NO_2_ | 2 |
| TMP | 0.15 | -0.15 | 0.01168 | 321 | C_10_H_13_N_2_O_8_P | 10 |
| dAMP | 0.14 | -0.15 | 0.011395 | 330 | C_10_H_12_N_5_O_6_P | 10 |
| AMP | 0.13 | -0.13 | 0.010825 | 346 | C_10_H_12_N_5_O_7_P | 10 |
| dGMP | 0.13 | -0.13 | 0.010825 | 346 | C_10_H_12_N_5_O_7_P | 10 |
| dCMP | 0.13 | -0.13 | 0.01225 | 306 | C_9_H_12_N_3_O_7_P | 9 |
| GMP | 0.13 | -0.13 | 0.010256 | 362 | C_10_H_12_N_5_O_8_P | 10 |
| CMP | 0.12 | -0.12 | 0.01168 | 322 | C_9_H_12_N_3_O_8_P | 9 |
| UMP | 0.12 | -0.12 | 0.01168 | 323 | C_9_H_11_N_2_O_9_P | 9 |
| Cysteine | 0.11 | -0.11 | 0.0316213 | 121 | C_3_H_7_NO_2_S | 3 |
| Lysine | 0.08 | -0.08 | 0.0227902 | 147 | C_6_H_15_N_2_O_2_ | 6 |
| Tryptophan | 0.07 | -0.07 | 0.0042732 | 204 | C_11_H_12_N_2_O_2_ | 11 |
| Methionine | 0.03 | -0.03 | 0.0059824 | 149 | C_5_H_11_NO_2_S | 5 |
| Proline | 0.00 | 0.00 | 8.831E-05 | 114 | C_5_H_9_NO_2_ | 5 |
| Ornithine | 0.00 | 0.00 | 5.413E-05 | 133 | C_5_H_13_N_2_O_2_ | 5 |
| Glucose | 0.00 | 0.00 | 0 | 180 | C_6_H_12_O_6_ | 6 |
| Sucrose | 0.00 | 0.00 | 0 | 342 | C_12_H_22_O_11_ | 12 |
| Fructose | 0.00 | 0.00 | 0 | 180 | C_6_H_12_O_6_ | 6 |
| Trehalose | 0.00 | 0.00 | 0 | 342 | C_12_H_22_O_11_ | 12 |
| Succinate | 0.00 | 0.00 | 0 | 116 | C_4_H_4_O_4_ | 4 |
| Fumarate | 0.00 | 0.00 | 0 | 114 | C_4_H_2_O_4_ | 4 |
| Malate | 0.00 | 0.00 | 0 | 132 | C_4_H_4_O_5_ | 4 |
| Shikimate | 0.00 | 0.00 | 0 | 173 | C_7_H_9_O_5_ | 7 |
| Urea | 0.00 | 0.00 | 0 | 60 | CH_4_N_2_O | 1 |
