# Supplementary material for: Flux Balance Analysis of Plant Metabolism: The Effect of Biomass Composition and Model Structure on Model Predictions
Source: Front Plant Sci. 2016 Apr 26;7:537. doi: 10.3389/fpls.2016.00537 (PMC4845513; doi:10.3389/fpls.2016.00537)
Supplement: Supplementary file 3 [file Table3.DOCX]

Table S3. Influence of individual biomass component on the growth rate predicted from AraGEM model in the ‘AraGEM-AraGEMBOF’ scenario. The growth rate was predicted by varying single biomass components up or down by 30%.

| **Compound** | **% Change in growth rate** | | **Coefficient** | **MW** | **Empirical formula** | **C atoms** |
| --- | --- | --- | --- | --- | --- | --- |
|  | **-30%** | **30%** |  |  |  |  |
| Starch | 3.94 | -3.65 | 0.470466 | 180 | C_6_H_12_O_6_ | 6 |
| Glutamate | 2.43 | -2.31 | 0.692383 | 146 | C_5_H_8_NO_4_ | 5 |
| Glutamine | 2.25 | -2.16 | 0.62137 | 146 | C_5_H_10_N_2_O_3_ | 5 |
| Cellulose | 2.23 | -2.13 | 0.541479 | 180 | C_6_H_12_O_6_ | 6 |
| Asparagine | 1.94 | -1.87 | 0.692383 | 132 | C_4_H_8_N_2_O_3_ | 4 |
| Sucrose | 1.37 | -1.33 | 0.170433 | 342 | C_12_H_22_O_11_ | 12 |
| Leucine | 1.32 | -1.29 | 0.248548 | 131 | C_6_H_13_NO_2_ | 6 |
| Phenylalanine | 1.05 | -1.03 | 0.142027 | 165 | C_9_H_11_NO_2_ | 9 |
| Serine | 0.83 | -0.81 | 0.372822 | 105 | C_3_H_7_NO_3_ | 3 |
| Valine | 0.75 | -0.74 | 0.177534 | 117 | C_5_H_11_NO_2_ | 5 |
| Aspartate | 0.72 | -0.71 | 0.287605 | 132 | C_4_H_6_NO_4_ | 4 |
| Palmitate | 0.69 | -0.68 | 0.046159 | 256 | C_16_H_32_O_2_ | 16 |
| Isoleucine | 0.63 | -0.63 | 0.10652 | 131 | C_6_H_13_NO_2_ | 6 |
| Tryptophan | 0.61 | -0.60 | 0.071014 | 204 | C_11_H_12_N_2_O_2_ | 11 |
| Threonine | 0.52 | -0.51 | 0.152679 | 119 | C_4_H_9_NO_3_ | 4 |
| Alanine | 0.45 | -0.45 | 0.189962 | 89 | C_3_H_7_NO_2_ | 3 |
| Glycine | 0.40 | -0.39 | 0.248548 | 75 | C_2_H_5_NO_2_ | 2 |
| Fructose | 0.38 | -0.38 | 0.094093 | 180 | C_6_H_12_O_6_ | 6 |
| Tyrosine | 0.37 | -0.37 | 0.05326 | 181 | C_9_H_11_NO_3_ | 9 |
| Glucose | 0.37 | -0.37 | 0.094093 | 180 | C_6_H_12_O_6_ | 6 |
| Cysteine | 0.09 | -0.09 | 0.042608 | 121 | C_3_H_7_NO_2_S | 3 |
| Proline | 0.07 | -0.07 | 0.017753 | 114 | C_5_H_9_NO_2_ | 5 |
| GMP | 0.05 | -0.05 | 0.006924 | 362 | C_10_H_12_N_5_O_8_P | 10 |
| AMP | 0.05 | -0.05 | 0.005148 | 346 | C_10_H_12_N_5_O_7_P | 10 |
| dAMP | 0.04 | -0.04 | 0.004793 | 330 | C_10_H_12_N_5_O_6_P | 10 |
| TMP | 0.04 | -0.04 | 0.004971 | 321 | C_10_H_13_N_2_O_8_P | 10 |
| CMP | 0.03 | -0.03 | 0.005859 | 322 | C_9_H_12_N_3_O_8_P | 9 |
| UMP | 0.03 | -0.03 | 0.005148 | 323 | C_9_H_11_N_2_O_9_P | 9 |
| dGMP | 0.02 | -0.02 | 0.003018 | 346 | C_10_H_12_N_5_O_7_P | 10 |
| dCMP | 0.02 | -0.02 | 0.003373 | 306 | C_9_H_12_N_3_O_7_P | 9 |
| Methionine | 0.02 | -0.02 | 0.003551 | 149 | C_5_H_11_NO_2_S | 5 |
| Lysine | 0.01 | -0.01 | 0.001775 | 147 | C_6_H_15_N_2_O_2_ | 6 |
| Arginine | 0 | 0 | 0 | 175 | C_6_H_15_N_4_O_2_ | 6 |
| Histidine | 0 | 0 | 0 | 155 | C_6_H_9_N_3_O_2_ | 6 |
| Ornithine | 0 | 0 | 0 | 133 | C_5_H_13_N_2_O_2_ | 5 |
| Trehalose | 0 | 0 | 0 | 342 | C_12_H_22_O_11_ | 12 |
| Succinate | 0 | 0 | 0 | 116 | C_4_H_4_O_4_ | 4 |
| Fumarate | 0 | 0 | 0 | 114 | C_4_H_2_O_4_ | 4 |
| Malate | 0 | 0 | 0 | 132 | C_4_H_4_O_5_ | 4 |
| Shikimate | 0 | 0 | 0 | 173 | C_7_H_9_O_5_ | 7 |
| Urea | 0 | 0 | 0 | 60 | CH_4_N_2_O | 1 |
