# Supplementary material for: Flux Balance Analysis of Plant Metabolism: The Effect of Biomass Composition and Model Structure on Model Predictions
Source: Front Plant Sci. 2016 Apr 26;7:537. doi: 10.3389/fpls.2016.00537 (PMC4845513; doi:10.3389/fpls.2016.00537)
Supplement: Supplementary file 4 [file Table4.DOCX]

Table S4. Influence of individual biomass component on the growth rate predicted from AraCore model in the ‘AraCore-AraCoreBOF’ scenario. The growth rate was predicted by varying single biomass components up or down 30%.

| **Compound** | **% Change in growth rate** | | **Coefficient** | **MW** | **Empirical formula** | **C atoms** |
| --- | --- | --- | --- | --- | --- | --- |
|  | **-30%** | **30%** |  |  |  |  |
| Cellulose | 5.42 | -4.89 | 0.652598 | 342 | C_12_H_22_O_11_ | 12 |
| M-ACP^[1]^ | 3.89 | -6.79 | 1.398423 | 118 | C_3_H_2_O_3_SR | 3 |
| Starch | 3.54 | -3.31 | 0.406977 | 342 | C_12_H_22_O_11_ | 12 |
| Leucine | 1.35 | -1.32 | 0.254585 | 131 | C_6_H_13_NO_2_ | 6 |
| Valine | 0.82 | -0.81 | 0.234863 | 117 | C_5_H_11_NO_2_ | 5 |
| Lysine | 0.78 | -0.77 | 0.166735 | 147 | C_6_H_15_N_2_O_2_ | 6 |
| Isoleucine | 0.76 | -0.75 | 0.145221 | 131 | C_6_H_13_NO_2_ | 6 |
| Phenylalanine | 0.75 | -0.74 | 0.107571 | 165 | C_9_H_11_NO_2_ | 9 |
| Threonine | 0.71 | -0.70 | 0.224106 | 119 | C_4_H_9_NO_3_ | 4 |
| Sucrose | 0.64 | -0.63 | 0.080678 | 342 | C_12_H_22_O_11_ | 12 |
| Arginine | 0.63 | -0.63 | 0.114742 | 175 | C_6_H_15_N_4_O_2_ | 6 |
| Alanine | 0.62 | -0.61 | 0.37112 | 89 | C_3_H_7_NO_2_ | 3 |
| Proline | 0.60 | -0.59 | 0.173906 | 114 | C_5_H_9_NO_2_ | 5 |
| Glutamate | 0.51 | -0.51 | 0.184664 | 146 | C_5_H_8_NO_4_ | 5 |
| Tyrosine | 0.50 | -0.49 | 0.0753 | 181 | C_9_H_11_NO_3_ | 9 |
| Glycine | 0.37 | -0.36 | 0.441041 | 75 | C_2_H_5_NO_2_ | 2 |
| Serine | 0.33 | -0.33 | 0.238449 | 105 | C_3_H_7_NO_3_ | 3 |
| Aspartate | 0.31 | -0.31 | 0.163149 | 132 | C_4_H_6_NO_4_ | 4 |
| Histidine | 0.31 | -0.31 | 0.053786 | 155 | C_6_H_9_N_3_O_2_ | 6 |
| Fumarate | 0.27 | -0.27 | 0.139842 | 114 | C_4_H_2_O_4_ | 4 |
| Methionine | 0.26 | -0.26 | 0.053786 | 149 | C_5_H_11_NO_2_S | 5 |
| Tryptophan | 0.24 | -0.24 | 0.026893 | 204 | C_11_H_12_N_2_O_2_ | 11 |
| Glutamine | 0.24 | -0.24 | 0.082471 | 146 | C_5_H_10_N_2_O_3_ | 5 |
| Asparagine | 0.24 | -0.24 | 0.111157 | 132 | C_4_H_8_N_2_O_3_ | 4 |
| Glucose | 0.23 | -0.23 | 0.060957 | 180 | C_6_H_12_O_6_ | 6 |
| Malate | 0.13 | -0.13 | 0.068128 | 132 | C_4_H_4_O_5_ | 4 |
| Fructose | 0.11 | -0.11 | 0.026893 | 180 | C_6_H_12_O_6_ | 6 |
| Ornithine | 0.10 | -0.10 | 0.026893 | 133 | C_5_H_13_N_2_O_2_ | 5 |
| Cysteine | 0.03 | -0.03 | 0.0502 | 121 | C_3_H_7_NO_2_S | 3 |
| Urea | 0.02 | -0.02 | 0.010757 | 60 | CH_4_N_2_O | 1 |
| dAMP | 0.02 | -0.02 | 0.001255 | 330 | C_10_H_12_N_5_O_6_P | 10 |
| Trehalose | 0.01 | -0.01 | 0.001793 | 342 | C_12_H_22_O_11_ | 12 |
| AMP | 0.01 | -0.01 | 0.001076 | 346 | C_10_H_12_N_5_O_7_P | 10 |
| TMP | 0.01 | -0.01 | 0.001255 | 321 | C_10_H_13_N_2_O_8_P | 10 |
| dGMP | 0.01 | -0.01 | 0.000717 | 346 | C_10_H_12_N_5_O_7_P | 10 |
| UMP | 0.01 | -0.01 | 0.001076 | 323 | C_9_H_11_N_2_O_9_P | 9 |
| GMP | 0.01 | -0.01 | 0.000717 | 362 | C_10_H_12_N_5_O_8_P | 10 |
| CMP | 0.01 | -0.01 | 0.000896 | 322 | C_9_H_12_N_3_O_8_P | 9 |
| dCMP | 0.01 | -0.01 | 0.000717 | 306 | C_9_H_12_N_3_O_7_P | 9 |
| Succinate | 0.00 | 0.00 | 0.001793 | 116 | C_4_H_4_O_4_ | 4 |
| Shikimate | 0.00 | 0.00 | 0.000538 | 173 | C_7_H_9_O_5_ | 7 |

^[1]^ M-ACP is malonyl-acyl carrier protein, the representative of lipid.
