# Supplementary material for: Flux Balance Analysis of Plant Metabolism: The Effect of Biomass Composition and Model Structure on Model Predictions
Source: Front Plant Sci. 2016 Apr 26;7:537. doi: 10.3389/fpls.2016.00537 (PMC4845513; doi:10.3389/fpls.2016.00537)
Supplement: Supplementary file 5 [file Table5.DOCX]

Table S5. Influence of maintenance on the growth rate predicted from Poolman model in the ‘Poolman-PoolmanBOF’ scenario, AraGEM model in the ‘AraGEM-AraGEMBOF’ scenario, and AraCore model in the ‘AraCore-AraCoreBOF’ scenario, respectively.

|  | **% Change in growth rate** | | |
| --- | --- | --- | --- |
|  | No maintenance | GAM | NGAM |
| Poolman-PoolmanBOF | 0 | 0 | 0 |
| AraGEM-AraGEMBOF | 29.59 | 0.66 | 28.75 |
| AraCore-AraCoreBOF | 1.34 | 0.57 | 1.34 |
