# Supplementary figures and images for: Flux Balance Analysis of Plant Metabolism: The Effect of Biomass Composition and Model Structure on Model Predictions
Source: Front Plant Sci. 2016 Apr 26;7:537. doi: 10.3389/fpls.2016.00537 (PMC4845513; doi:10.3389/fpls.2016.00537)

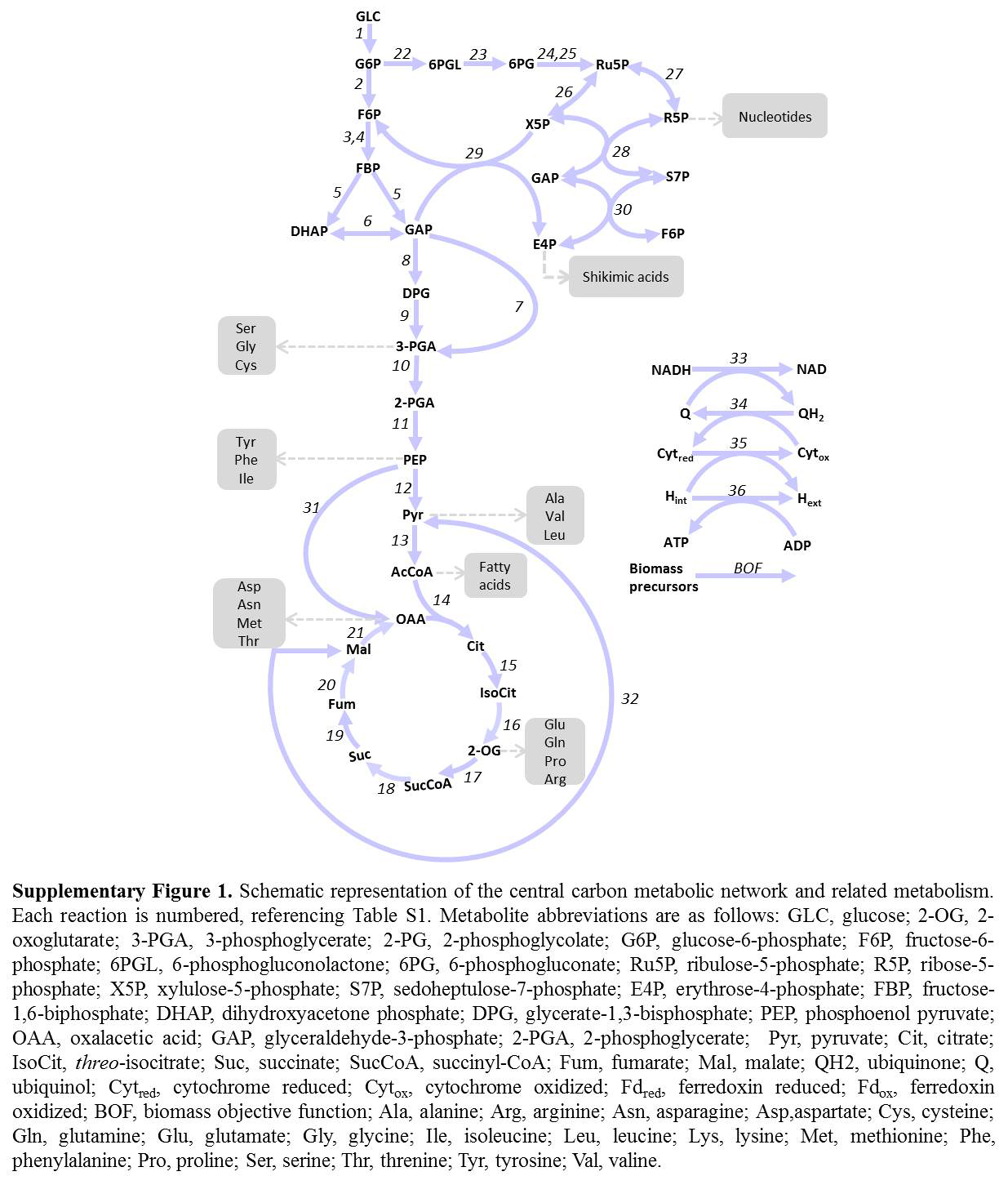

Supplement: Supplementary file 10 [file Image1.JPEG]

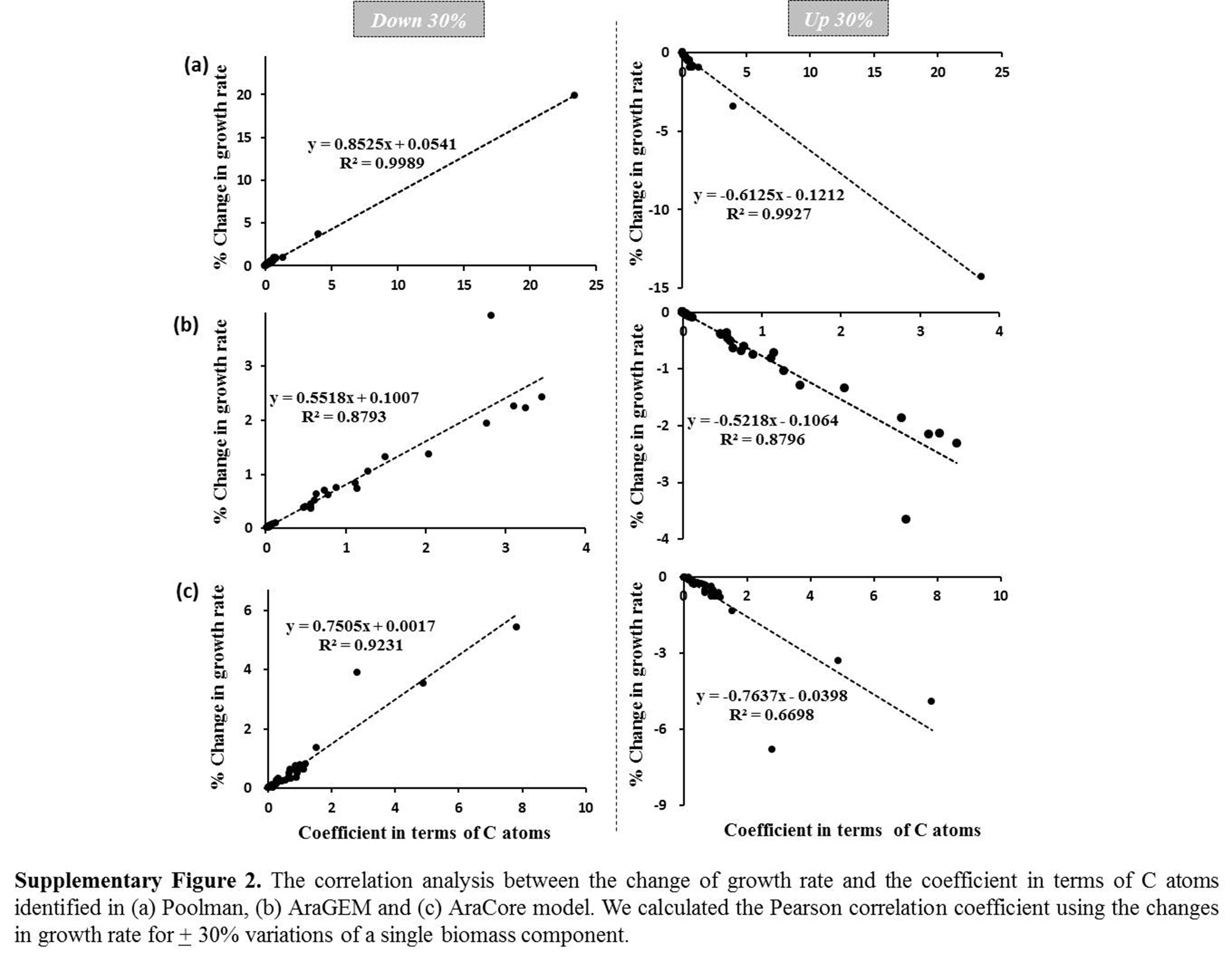

Supplement: Supplementary file 11 [file Image2.JPEG]

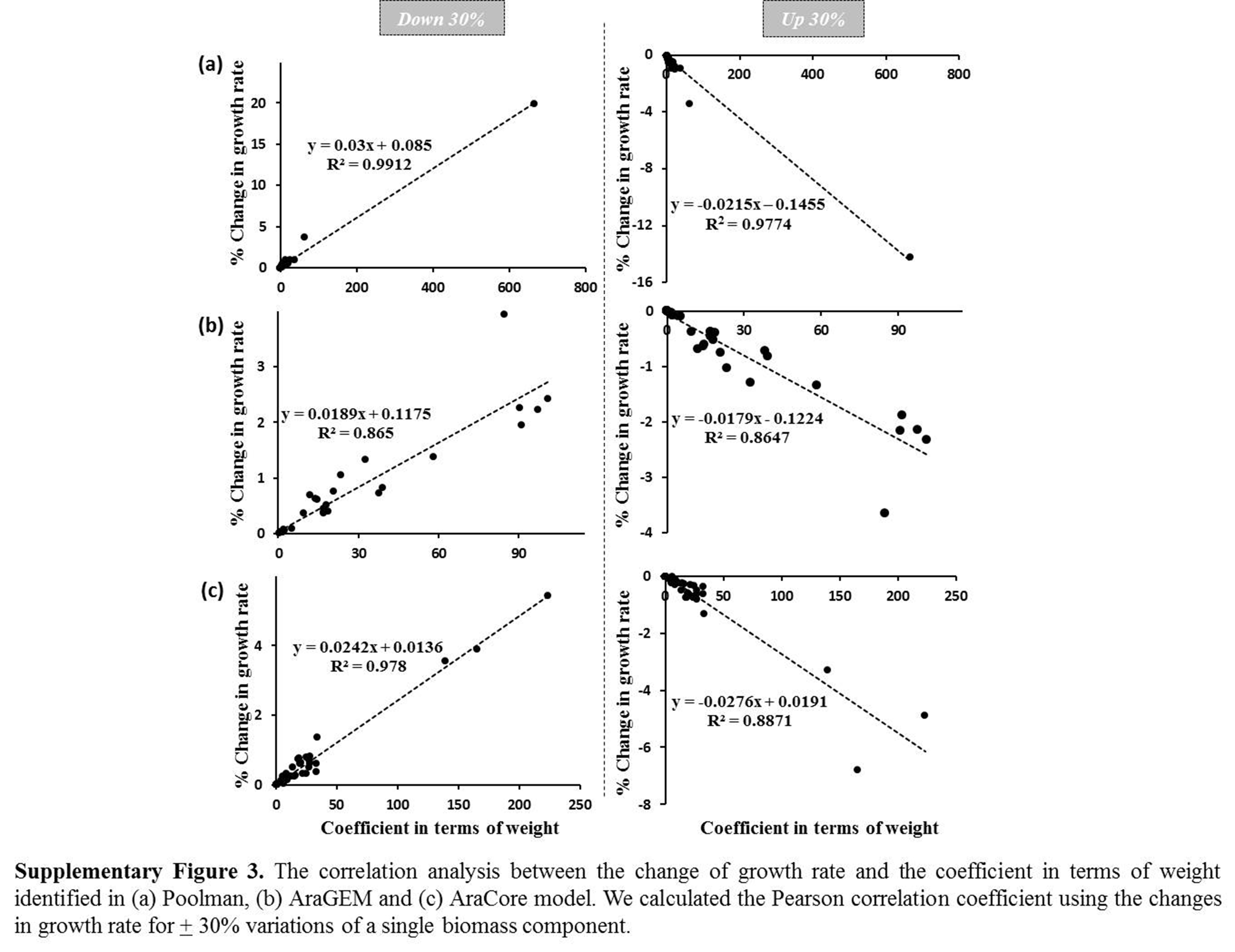

Supplement: Supplementary file 12 [file Image3.JPEG]
